# Clinical Efficacy of Antianlotinib Combined with Immune Checkpoint Inhibitors in the Treatment of Advanced Non-Small-Cell Lung Cancer and Its Effect on Serum VEGF, CEA, and SCC-Ag

**DOI:** 10.1155/2022/1530875

**Published:** 2022-10-14

**Authors:** Linghui He, Xi Chen, Lingchi Ding, Xuedong Zhang

**Affiliations:** ^1^Department of Medical Oncology, Tumor Hospital Affiliated to Nantong University & Nantong Tumor Hospital, Nantong 226000, China; ^2^Department of Thoracic Surgery, The Sixth People's Hospital of Nantong, Affiliated Nantong Hospital of Shanghai University, Nantong 226011, China

## Abstract

**Purpose:**

This study is aimed at investigating the clinical safety and effectiveness of anlotinib combined with immune checkpoint inhibitors (ICIs) in the treatment of non-small-cell lung cancer (NSCLC).

**Methods:**

We selected 68 NSCLC patients treated at the Tumor Hospital Affiliated to Nantong University from October 2019 to January 2022. Patients receiving ICI monotherapy were included in the control group (*n* = 36), whereas patients receiving anlotinib combined with ICIs were enrolled in the study group (*n* = 32). The survival, adverse reactions (AEs), and short-term clinical effectiveness of the two groups were observed. The tumor markers (vascular endothelial growth factor (VEGF), carcino-embryonic antigen (CEA), and squamous cell carcinoma antigen (SCC-AG)) and T lymphocyte subsets (CD3^+^, CD4^+^, CD8^+^, and CD4^+^/CD8^+^) were determined before and after treatment.

**Results:**

Compared with the control group, the disease-control rate (DCR) and objective response rate (ORR) in the study group were substantially higher than that of the control group (62.50 vs. 36.11, 81.25 vs. 55.56; *P* < 0.05). The serum levels of VEGF, CEA, and SCC-AG in the two groups were considerably lower after two cycles of treatment (*P* < 0.05), and the serum levels of VEGF, CEA, and SCC-AG in the study group were significantly lower than those in the control group (*P* < 0.05). Following therapy, CD8^+^ in both groups decreased dramatically (*P* < 0.05), whereas CD3^+^, CD4^+^, and CD4^+^/CD8^+^ were significantly increased, but there was no statistical difference between the two groups (*P* > 0.05). The incidence of gastrointestinal, respiratory, cardiovascular, and immune-related adverse events did not significantly differ between the two groups (*P* > 0.05). The median progression-free survival (PFS) in the control and study groups for the first-line treatment patients was 7.2 and 9.8 months, respectively, whereas for the second-line treatment patients, it was 4.2 and 6.4 months, respectively. The mean PFS of study group was substantially longer than that of the control group regardless of the first- or second-line treatment. According to Cox analysis, the number of drug lines and TNM stage was independent risk variables impacting the prognosis of patients in this study.

**Conclusion:**

The combination of anlotinib with ICIs was more effective than either agent alone in the first- and second-line treatment of patients with advanced NSCLC. This treatment regimen did not interfere with immunological recovery or increase side effects.

## 1. Introduction

Non-small-cell lung cancer (NSCLC) is one of the most prevalent malignancies and accounts for 85% of all lung cancers [1]. Clinically, the prognosis of NSCLC is still not ideal. Research has shown that patients with stage-IB NSCLC have a 60-month overall survival rate of around 68%, whereas those with stage IVA–IVB NSCLC have a 60-month overall survival rate of less than 10% [2]. A small percentage of NSCLC patients are detected at an early stage (stage I or II) when they may be treated with surgical resection and have a better prognosis. At the time of diagnosis, more than 60% of lung cancer patients have locally progressed disease or metastatic disease (stage III or IV). At this time, most patients cannot undergo surgical resection and can be treated only with conventional chemotherapy and radiotherapy [3].

Advances in our understanding of how tumor cells evade the immune system have led to the development of immunotherapy strategies, most notably immune checkpoint inhibitors (ICIs), which show effectiveness against advanced NSCLC. Thus, they have become a new hope for cancer patients after multiline therapy failure. However, ICI monotherapy is prone to drug resistance, and researchers are actively exploring multidrug combination immunotherapy to delay or prevent the occurrence of drug resistance to ICIs [4]. Anlotinib is an antitumor drug newly approved for clinical use. It prevents tumor growth by inhibiting signaling pathways involved in angiogenesis and cell survival [5]. Anlotinib shows promising clinical effectiveness in the treatment of NSCLC in phase II and III clinical studies [6, 7]. At present, anlotinib has been reported as a promising new first-line treatment strategy for NSCLC patients. Chu et al. enrolled non-small-cell lung cancer patients without EGFR/ALK/ROS1 mutations, who are given sintilimab and anlotinib as the first-line treatment. The results showed that the objective response rate was 72.7%, and the disease control rate was 100% [8]. Nevertheless, the clinical effectiveness of anlotinib combined with ICIs for the first- and second-line therapy of NSCLC is unclear, and few trials have been conducted in the United States. In the present study, we evaluate the comparative clinical effectiveness and safety of anlotinib combined with ICIs with ICI monotherapy in patients with advanced NSCLC as the first- and second-line treatment.

## 2. Material and Methods

### 2.1. Baseline Data

A total of 68 NSCLC patients with a median age of 60 and an average age of 57.85 ± 10.58 years who received care at our institution between October 2019 and January 2022 were selected. All patients were grouped according to the administration of medication. Patients receiving ICI monotherapy were selected as the control group (*n* = 36), and patients receiving anlotinib combined with ICIs were selected as the study group (*n* = 32). All included patients submitted written informed consents. The baseline information for the two patient groups was similar, as demonstrated in Table 1 (*P* > 0.05). This study was approved by the Ethics Committee of Nantong Tumor Hospital (NO. 2021-059).

The inclusion criteria were as follows: (1) imaging and pathology-diagnosed NSCLC, and patients with histological type adenocarcinoma were required to have a negative driver gene (EGFR or ALK) with PD-L1 test ≥ 1%. Measurable lesions were evaluated by imaging. (2) Patients were not treated with radical surgery; (3) no ICIs were received; (4) a 3-month survival time was projected; and (5) at least two cycles or more of ICIs alone or anlotinib combined with ICIs were received. The exclusion criteria were as follows: (1) allergic or contraindicated to anlotinib or ICIs; (2) patients with malignant tumors in other parts of the body; (3) heart, lung, and kidney inadequate patients; and (4) pregnant or lactating women.

### 2.2. Treatment

For the control group, the selected patients received ICI monotherapy and were given 2 mg/kg pembrolizumab intravenous infusion, with each infusion time being more than 0.5 h. Infusion was once every 3 weeks, and the treatment was continued for 3 months.

For the study group, the observation group received 12 mg of oral anlotinib plus those used in the control group once a day for 2 consecutive weeks and then rested for 1 week. The total of treatment time is 3 months. If the patient acquired disease progression, serious adverse reactions (AEs), or drug toxicity, the drug should be discontinued immediately.

### 2.3. Evaluation of Efficacy, AEs, and Survival

Disease-control rate (DCR) and objective response rate (ORR) were used to determine the treatment efficacy. The following methodology was used in the calculations. Complete remission (CR): all tumor lesions disappeared, and no new lesions appeared for at least four weeks. Partial remission (PR): the sum of the maximum diameters of tumor lesions was reduced by ≥30% for at least four weeks. Stable disease (SD) was defined as a drop of <30% or an increase of >20%. Progression of disease (PD) was defined as an increase of ≥20% in the sum of the maximum diameters of the lesions compared with prior therapy or the identification of metastatic lesions. DCR (%) = CR% + PR% + SD%and ORR (%) = CR% + PR%. AEs were evaluated according to CTCAE5.0.

The survival of patients was collected by telephone follow-up, and their progression-free survival (PFS) was calculated with statistical software.

### 2.4. Observation Indicators

Blood samples were collected from all patients one day before treatment and 3 months after treatment. The serum levels of VEGF, CEA, and SCC-Ag were detected using ELISA kits, and the levels of T lymphocyte subsets (CD3^+^, CD4^+^, CD8^+^, and CD4^+^/CD8^+^) were determined using flow cytometry before and after therapy.

### 2.5. Statistical Analysis

Statistical analysis and visualization of data were performed using SPSS and GraphPad Prism 8 software. Measurement data are expressed as “*x* ± SD.” To analyze the enumeration data, presented as a percentage, we used the *t*-test and *χ*^2^ test. The K-M approach was used to create a survival curve, and the COX method was used to identify and eliminate potential predictors of poor outcomes. Statistically significant difference was set at the *P* < 0.05 level.

## 3. Results

### 3.1. Clinical Efficacy

Compared with the control group, the ORR and DCR in the study group were statistically higher than that of the control group (62.50 vs. 36.11 and 81.25 vs. 55.56), respectively (*P* < 0.05). Compared with the first- and second-line treatment, no statistically significant difference was found in ORR or DCR (*P* > 0.05). The details are provided in Table 2.

### 3.2. The Change of Tumor Markers and T Lymphocyte Subsets before and after Treatment

**Figure 1:**
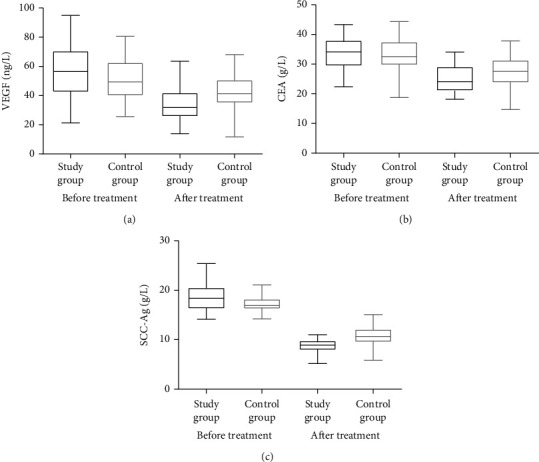
Changes in tumor marker levels.

**Figure 2:**
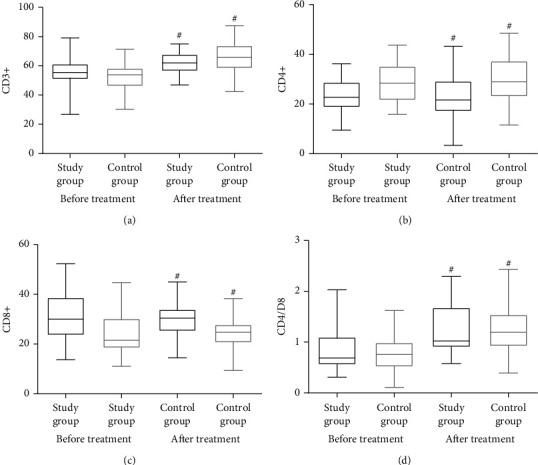
Changes in the levels of T lymphocyte subsets. ^#^*P* < 0.05 vs. before treatment.

It was found that there was not a single indicator differed significantly in the study group and control group (*P* > 0.05). However, the serum levels of VEGF, CEA, and SCC-Ag were considerably lower (*P* < 0.05) after two cycles of therapy in both groups. A statistically significant difference also existed between the study and the control group in terms of VEGF, CEA, and SCC-Ag levels, with the study group having much lower levels overall. After receiving therapy, both groups showed increases in CD3^+^, CD4^+^, and CD4^+^/CD8^+^ T lymphocytes, but CD8^+^ T h = lymphocytes decreased considerably (*P* < 0.05). There was no significant difference existed between the two groups (*P* > 0.05) (Table 3 and Figures 1 and 2).

### 3.3. Adverse Events (AEs)

**Figure 3:**
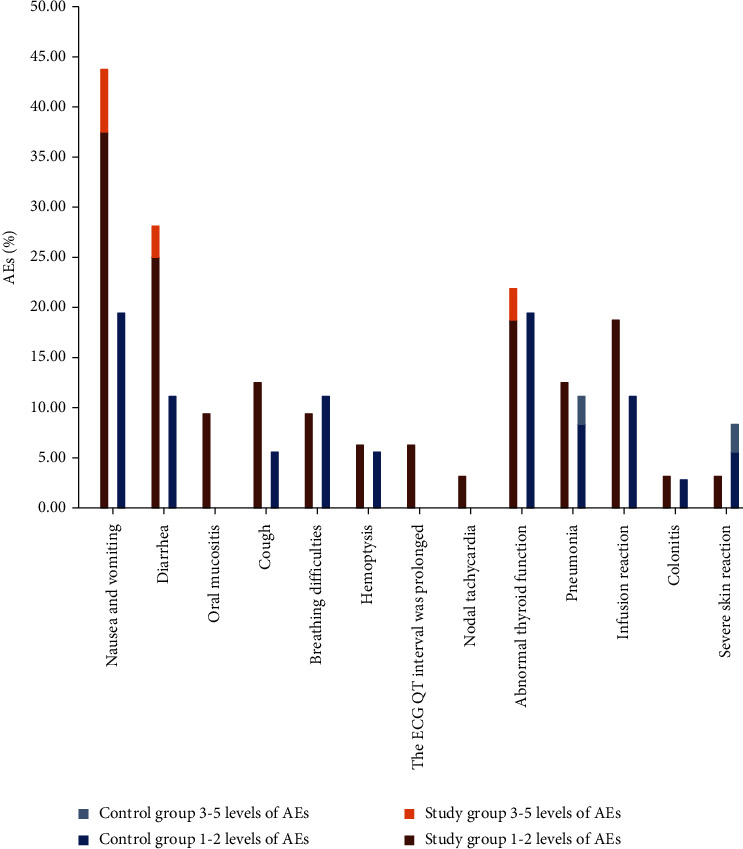
Occurrence of adverse effects (AEs).

AEs in the control and study groups primarily occurred as immune-related AEs and gastrointestinal system-related AEs. There was a trend toward more gastrointestinal AEs in the study group compared with the control group, but the difference was not statistically significant (*P* > 0.05) as shown in Table 4 and Figure 3.

### 3.4. PFS Analysis

**Figure 4:**
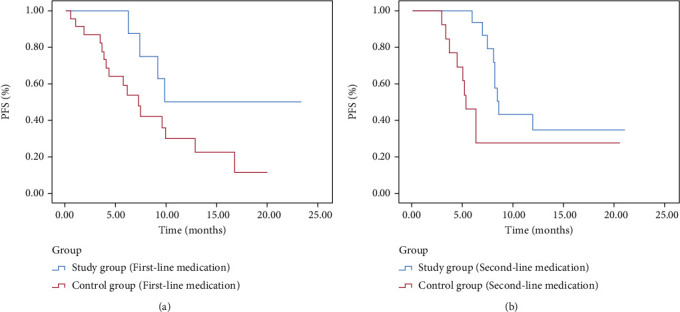
PFS analysis.

In the control and study groups, the median PFS was 7.2 and 9.8 months in patients with the first-line treatment in the study group and control group, respectively. The median PFS was 4.2 and 6.4 months in patients with the second-line treatment in the study group and control group, respectively. Regardless of the first- or second-line drugs, the study group had a considerably longer median PFS than the control group (15.75 ± 2.73 vs. 8.95 ± 1.51 months and 10.13 ± 1.35 vs. 7.28 ± 1.65 months, respectively; *P* < 0.05) (Table 5 and Figure 4).

### 3.5. Cox Analysis

Cox analysis results are shown in Tables 6 and 7. The independent risk variables impacting patients' prognoses in this research were the number of drug lines and TNM stage (*P* < 0.05).

## 4. Discussion

The development of immunotherapy has led to a shift in the approach to treating advanced NSCLC owing to the development of ICIs that target the PD-1/PD-L1 pathway. ICIs targeting the PD-1/PD-L1 pathway primarily include nivolumab, atezolizumab, and pembrolizumab. For individuals with NSCLC, the ICI pembrolizumab is presently the primary choice. Foreign researchers have reported that in patients with previously untreated and driver-gene-negative metastatic nonsquamous NSCLC, when added to regular chemotherapy, pembrolizumab dramatically increases the overall survival and PFS compared with chemotherapy alone [9]. Goldberg et al. [10] illustrated that pembrolizumab has a good curative effect on NSCLC patients with brain metastases with PD-L1 ≥ 1%, and no serious AEs occur during treatment, indicating its safety and effectiveness.

In addition to the PD-1/PD-L1 pathway, NSCLC therapy relies heavily on several different molecular mechanisms [11]. To interact with cell growth factors and external ligands, cells use transmembrane glycoproteins called receptor tyrosine kinases (RTKs). RTKs mediate many important physiological processes, including cell proliferation, growth, migration, differentiation, and apoptosis. It is linked to many different medical problems including cancer and heart disease. Anlotinib is a popular medicine for NSCLC treatment. It is a new oral tyrosine kinase inhibitor that targets receptors like VEGF to suppress tumor angiogenesis and growth [12]. Wang et al. [13] and other studies have shown that anlotinib in the treatment of NSCLC has an antiangiogenic effect better than those of three main clinical antiangiogenic drugs (sunitinib, sorafenib, and nintedanib). In terms of AEs, compared with sunitinib, anlotinib has a lower incidence of grade ≥ 3 AEs [13]. As a result of its recent introduction to the Chinese market in 2018, clinical data on the effectiveness and safety of pembrolizumab combined with anlotinib for the treatment of NSCLC patients are limited. Chen et al. [14] reported that pembrolizumab combined with anlotinib improves survival in patients previously treated with EGFR-mutant NSCLC.

In the present study, patients with advanced NSCLC were administered with the anlotinib + pembrolizumab as the first or secondary therapy and then compared with pembrolizumab monotherapy. Results showed that compared with monotherapy, the combination of the two drugs significantly improved the ORR and DCR. The VEGF, CEA, and SCC-Ag are common tumor markers, and their levels in serum can reflect the tumor burden to a certain extent and be used for efficacy evaluation. There was no significant difference in baseline markers between the two groups in this research. Three tumor indicators (VEGF, CEA, and SCC-Ag) were substantially reduced in the study group after two cycles of therapy compared with the control group. The combined use of the two drugs was more effective than the single-drug treatment. Results of survival analysis also showed that the combination of the two drugs prolonged the PFS regardless of whether they were used as first- or second-line treatment. The median PFS was 7.2 and 9.8 months in the patients with first-line treatment in the control and study groups, respectively, and the mean PFS was 8.95 ± 1.51 and 15.75 ± 2.73 months, respectively. The median PFS of the patients with the second-line treatment in the control and study groups was 4.2 and 6.4 months, respectively, and the mean PFS was 7.28 ± 1.65 and 10.13 ± 1.35 months, respectively. COX analysis revealed that the drug line and TNM stage were significant prognostic variables. Based on these findings, more clinical attention should be paid to the use of ICIs in conjunction with anlotinib for the treatment of advanced NSCLC patients under the first- and second-line conditions.

The human body's cellular immunity is considered to be the main antitumor immunity. The massive infiltration of lymphocytes is the core of the antitumor response of cellular immunity, and it is also the cytological basis for the effect of immunotherapy. Accordingly, the monitoring of lymphocyte subsets has guiding significance for evaluating tumor drug efficacy. A combination of treatments increased the proportion of CD3^+^, CD4^+^, and CD4^+^/CD8^+^ while simultaneously decreasing the proportion of CD8^+^ cells. The results of this study were consistent with those of previous ones indicating that the functional damage of T lymphocyte subsets is reversible, and that it can return to normal when the tumor burden is reduced or relieved. Additionally, the combination of the two drugs can affect the recovery of immune function. Whether the combination of the two drugs increases AEs is a special concern in clinical practice. The present research has found that the most common AEs occurred during monotherapy were those linked to the immune system and the digestive system. The incidence of AEs related to the gastrointestinal system slightly increased when the two drugs were combined. However, compared with the results obtained using a single drug, no discernible difference existed. Apparently, the combination of ICIs and anlotinib was safe because the incidence of AEs was reduced.

In conclusion, using anlotinib combined with ICIs as the first- or second-line treatment is more effective than ICI monotherapy in the treatment of advanced NSCLC patients. Therefore, this combination has potential therapeutic applications.

## Figures and Tables

**Table 1:** Baseline data of patients.

Characteristics	Study group (*n* = 32)	Control group (*n* = 36)	*t*/*χ*^2^	*P*
Gender				
Male	16	14	0.8483	0.3570
Female	16	22		
Age (years)				
>60	19	19	0.2991	0.5845
≤60	13	17		
Smoking history				
Yes	18	21	0.0301	0.8623
No	14	15		
TNM stage				
IIIC	9	12	0.2153	0.6426
IV	23	24		
Number of tumor				
Solitary	7	6	0.2972	0.5856
Multiple	25	30		
Histological type				
Adenocarcinoma	20	22	0.2183	0.6404
Squamous cell carcinoma	10	14		
Line of medication				
First-line	13	23	3.680	0.0551
Second-line	19	13		
Brain metastases				
Yes	2	3	0.1079	0.7425
No	30	33		

**Table 2:** Comparison of clinical efficacy between the study and control groups of patients.

Group	CR (*n* (%))	PR (*n* (%))	SD (*n* (%))	PD (*n* (%))	ORR (*n* (%))	DCR (*n* (%))
Study group	3 (9.38)	17 (53.13)	6 (18.75)	6 (18.75)	20 (62.50)^∗^	26 (81.25)^∗^
Control group	1 (2.78)	12 (33.33)	7 (19.44)	16 (44.45)	13 (36.11)	20 (55.56)
First-line medication						
Study group	2 (6.25)	10 (31.25)	1 (3.13)	0 (0.00)	12 (37.50)	13 (40.63)
Control group	1 (2.78)	8 (22.22)	4 (11.11)	10 (27.78)	9 (25.00)	13 (36.11)
Second-line medication						
Study group	1 (3.13)	7 (18.75)	5 (15.63)	6 (18.75)	8 (25.00)	13 (40.63)
Control group	0 (0.00)	4 (11.11)	3 (8.33)	6 (16.67)	4 (11.11)	7 (19.44)

^∗^ *P* < 0.05, compared with the control group.

**Table 3:** T lymphocyte subsets and serum level of vascular endothelial growth factor before and after therapy.

Subject	Study group		Control group	
	Before treatment	After treatment	Before treatment	After treatment
VEGF (ng/L)	56.29 ± 18.58	34.20 ± 11.04^∗^^#^	51.20 ± 14.61	41.94 ± 12.89^#^
CEA (*μ*g/L)	33.64 ± 5.39	24.81 ± 4.47^∗^^#^	32.97 ± 6.06	27.55 ± 5.34^#^
SCC-Ag (*μ*g/L)	18.58 ± 2.64	8.79 ± 1.35^∗^^#^	17.24 ± 1.73	10.79 ± 2.23^#^
CD3^+^	55.77 ± 10.91	61.79 ± 7.72^#^	52.41 ± 8.76	65.59 ± 10.27^#^
CD4^+^	23.32 ± 8.09	28.46 ± 8.09^#^	22.61 ± 9.54	29.79 ± 9.72^#^
CD8^+^	30.71 ± 9.56	24.51 ± 8.41^#^	30.50 ± 7.58	23.81 ± 6.49^#^
CD4^+^/CD8^+^	0.83 ± 0.38	1.30 ± 0.66^#^	0.78 ± 0.36	1.42 ± 0.88^#^

^∗^ *P* < 0.05, compared with the control group; ^#^*P* < 0.05, compared with before treatment.

**Table 4:** Occurrence of adverse effects (AEs).

Types of AEs	Study group (*n* (%))		Control group (*n* (%))	
	Levels 1–2	Levels 3–5	Levels 1–2	Levels 3–5
Gastrointestinal system				
Nausea, vomit	12 (37.50)	2 (6.25)	8 (22.22)	0 (0.00)
Diarrhea	8 (25.00)	1 (3.13)	4 (11.11)	0 (0.00)
Oral mucositis	3 (9.38)	0 (0.00)	0 (0.00)	0 (0.00)
Respiratory system				
Cough	4 (12.50)	0 (0.00)	2 (5.56)	0 (0.00)
Difficult breathing	3 (9.38)	0 (0.00)	4 (11.11)	0 (0.00)
Hemoptysis	2 (6.25)	0 (0.00)	2 (5.56)	0 (0.00)
Cardiovascular system				
ECG QT prolongation	2 (6.25)	0 (0.00)	0 (0.00)	0 (0.00)
Sinus tachycardia	1 (3.13)	0 (0.00)	0 (0.00)	0 (0.00)
Immune-related AEs				
Thyroid dysfunction	6 (18.75)	1 (3.13)	7 (19.44)	0 (0.00)
Pneumonia	4 (12.50)	0 (0.00)	3 (8.33)	1 (2.78)
Transfusion reaction	6 (18.75)	0 (0.00)	4 (11.11)	0 (0.00)
Colitis	1 (3.13)	0 (0.00)	1 (2.78)	0 (0.00)
Severe skin reaction	1 (3.13)	0 (0.00)	2 (5.56)	1 (2.78)

^∗^ *P* < 0.05, compared with the control group.

**Table 5:** Results of univariable analysis of the number of drug lines and progression-free survival.

	Median PFS (month)	Average PFS (month)	95% CI	*P* value
First-line medication				
Study group	7.2	8.95 ± 1.51	5.99–11.91	0.0483
Control group	9.8	15.75 ± 2.73	10.40–21.10	
Second-line medication				
Study group	4.2	7.28 ± 1.65	4.05–10.51	0.0451
Control group	6.8	10.13 ± 1.35	7.49–12.77	

**Table 6:** COX univariate analysis.

Features	*B*	SE	Wald	Df	*P*	Exp(*B*)
Gender	-1.135	0.7157	2.514	1	0.1128	0.3214
Age	1.891	1.004	3.551	1	0.0595	6.629
Smoking history	-0.5602	0.7181	0.6085	1	0.4353	0.5711
TNM stage	3.812	1.175	10.53	1	0.0012	45.25
Number of tumors	1.320	0.9133	2.089	1	0.1483	3.744
Line of medication	2.122	0.9933	4.566	1	0.0326	8.352
Histology types	0.7421	1.000	0.5511	1	0.4579	2.101

**Table 7:** COX multivariate analysis.

Features	*B*	SE	Wald	Df	*P*	Exp(*B*)
Age	1.119	0.8461	3.791	1	0.3180	1.245
TNM stage	4.698	2.619	8.374	1	0.03915	60.94
Line of medication	3.347	1.614	7.051	1	0.04128	13.08

## Data Availability

All experimental data used to support the findings of this study are available from the corresponding author upon request.

## References

[1] Si X., Zhang L., Wang H. (2018). Quality of life results from a randomized, double-blinded, placebo-controlled, multi-center phase III trial of anlotinib in patients with advanced non-small cell lung cancer. *Lung Cancer*.

[2] Duma N., Santana-Davila R., Molina J. R. (2019). Non-small cell lung cancer: epidemiology, screening, diagnosis, and treatment. *Mayo Clinic Proceedings*.

[3] Osmani L., Askin F., Gabrielson E., Li Q. K. (2018). Current WHO guidelines and the critical role of immunohistochemical markers in the subclassification of non-small cell lung carcinoma (NSCLC): moving from targeted therapy to immunotherapy. *Seminars in Cancer Biology*.

[4] Schoenfeld A. J., Hellmann M. D. (2020). Acquired resistance to immune checkpoint inhibitors. *Cancer Cell*.

[5] Wang G., Sun M., Jiang Y. (2019). Anlotinib, a novel small molecular tyrosine kinase inhibitor, suppresses growth and metastasis via dual blockade of VEGFR2 and MET in osteosarcoma. *International Journal of Cancer*.

[6] Han B., Li K., Zhao Y. (2018). Anlotinib as a third-line therapy in patients with refractory advanced non-small-cell lung cancer: a multicentre, randomised phase II trial (ALTER0302). *British Journal of Cancer*.

[7] Han B., Li K., Wang Q. (2018). Effect of anlotinib as a third-line or further treatment on overall survival of patients with advanced non–small cell lung cancer: the ALTER 0303 phase 3 randomized clinical trial. *JAMA Oncology*.

[8] Chu T., Zhong R., Zhong H. (2021). Phase 1b study of sintilimab plus anlotinib as first-line therapy in patients with advanced NSCLC. *Journal of Thoracic Oncology*.

[9] Gandhi L., Rodríguez-Abreu D., Gadgeel S. (2018). Pembrolizumab plus chemotherapy in metastatic non-small-cell lung cancer. *The New England Journal of Medicine*.

[10] Goldberg S. B., Schalper K. A., Gettinger S. N. (2020). Pembrolizumab for management of patients with NSCLC and brain metastases: long-term results and biomarker analysis from a non-randomised, open-label, phase 2 trial. *The Lancet Oncology*.

[11] Liang L., Hui K., Hu C. (2019). Autophagy inhibition potentiates the anti-angiogenic property of multikinase inhibitor anlotinib through JAK2/STAT3/VEGFA signaling in non-small cell lung cancer cells. *Journal of Experimental & Clinical Cancer Research*.

[12] Gao Y., Liu P., Shi R. (2020). Anlotinib as a molecular targeted therapy for tumors. *Oncology Letters*.

[13] Wang W., Shao L., Xu Y. (2022). Efficacy and safety of anlotinib with and without EGFR-TKIs or immunotherapy in the treatment of elder patients with non-small-cell lung cancer: a retrospective study. *BMC Pulmonary Medicine*.

[14] Chen Y., Yang Z., Wang Y. (2021). Pembrolizumab plus chemotherapy or anlotinib vs. pembrolizumab alone in patients with previously treated EGFR-mutant NSCLC. *Frontiers in Oncology*.

